# Number of Teeth and Nutritional Status Parameters Are Related to Intima-Media Thickness in Dalmatian Kidney Transplant Recipients

**DOI:** 10.3390/jpm12060984

**Published:** 2022-06-16

**Authors:** Maja Dodig Novaković, Sanja Lovrić Kojundžić, Mislav Radić, Marijana Vučković, Andrea Gelemanović, Marija Roguljić, Katja Kovačević, Josip Orešković, Josipa Radić

**Affiliations:** 1 Department of Radiology, General Hospital Šibenik, 22000 Šibenik, Croatia; dodig.maja@gmail.com; 2 Department of Diagnostic and Interventional Radiology, University Hospital of Split, 21000 Split, Croatia; lovric.sanja@gmail.com; 3 School of Medicine, University of Split, 21000 Split, Croatia; 4 Department of Health Studies, University of Split, 21000 Split, Croatia; 5 Department of Internal Medicine, Division of Clinical Immunology and Rheumatology, University Hospital of Split, 21000 Split, Croatia; mislavradic@gmail.com; 6 Department of Internal Medicine, School of Medicine, University of Split, 21000 Split, Croatia; 7 Department of Nephrology and Dialysis, University Hospital of Split, 21000 Split, Croatia; mavuckovic@kbsplit.hr; 8 Biology of Robusteness Group, Mediterranean Institute for Life Sciences (MedILS), 21000 Split, Croatia; andrea.gelemanovic@gmail.com; 9 Department of Oral Medicine and Periodontology, School of Medicine, Study of Dental Medicine, University of Split, 21000 Split, Croatia; marija.roguljic@mefst.hr; 10 Private Dental Practice Split, 21000 Split, Croatia; katjakovacevic3@gmail.com; 11 Private Dental Practice Josip Orešković, 34000 Požega, Croatia; josipo555@gmail.com

**Keywords:** kidney transplant, periodontitis, atherosclerosis, nutritional status, body composition, advanced glycation end-products

## Abstract

Although kidney transplantation significantly improves the quality of life of patients with end-stage renal disease (ESRD), the prevalence of cardiovascular disease (CVD) in kidney transplant recipients (KTRs) remains high. Atherosclerosis, post-transplantation metabolic changes, immunosuppressive therapy, and periodontitis contribute to elevated cardiovascular risk in this population. The aim of the study was to evaluate carotid intima-media thickness (IMT) as a surrogate marker of atherosclerosis and to analyze the possible risk factors for IMT in Dalmatian KTRs. Ninety-three KTRs were included in this study. Data on clinical and laboratory parameters, body composition, anthropometry, advanced glycation end-product (AGE) measurements, blood pressure, and arterial stiffness were collected. All participants underwent ultrasound examination of IMT and evaluation of periodontal status. KTRs with carotid IMT ≥ 0.9 were significantly older, had a lower level of total cholesterol, fat mass, end-diastolic velocity (EDV), and had fewer teeth. They also had significantly higher values of pulse wave velocity (PWV) and resistive index (RI). We found positive correlations between carotid IMT and duration of dialysis, age, PWV, AGE, RI, and average total clinical attachment level (CAL). The regression model showed that IMT in KTRs is associated with higher PWV, lower fat mass, and fewer teeth. The results of our study suggest that nutritional and periodontal status are associated with carotid IMT in KTRs.

## 1. Introduction

Chronic kidney disease (CKD) and especially end-stage renal disease (ESRD) are known as great risk factors for cardiovascular (CV) events and complications [1]. Kidney transplantation (KTX) is the therapy of choice for the treatment of ESRD and offers multiple advantages for kidney transplant recipients (KTRs) [2]. Despite all mentioned advantages, KTRs still have a greater risk of developing major CVD and have a shorter life expectancy than the general population [3,4].

Atherosclerosis is believed to be one of the major contributors to elevated CV risk in the CKD population [5,6]. Mechanisms connecting ESRD and atherosclerosis include mineral metabolism disturbances affecting vascular calcification, chronic volume overload through effects on hypertension and hypertrophy of the left ventricle, effects of advanced glycation end-products (AGEs) through endothelial dysfunction [7], chronic infectious disease, and angiopoietin-2 effects [8]. After KTX, the effects of pretransplantation risk factors on atherosclerosis are diminutive, but the post-transplant period bears challenges such as immunosuppressants, weight gain, post-transplantation diabetes mellitus, and chronic infections, also contributing to atherosclerosis and arterial stiffness [8,9] in this patient population.

The sonographically measured intima-media thickness (IMT) is considered to represent a surrogate marker for atherosclerosis. The IMT measurement can be applied in clinical practice to detect early atherosclerosis and predict atherosclerotic plaque instability in a variety of populations [10]. Higher values of carotid IMT and femoral IMT are associated with higher rates and degrees of histopathological atherosclerosis in CKD patients [11].

The impact of KTR on carotid IMT is conflicting. One study demonstrated carotid IMT to progressively increase early (2, 4, and 6 months) after kidney transplantation [12], while, in contrast, another study reported improvements 6 months after transplant [13]. Despite these conflicting results, values of IMT in KTRs are often still higher compared to the general population [13,14].

The role of nutrition and nutritional status in the pathogenesis of atherosclerosis and CV risk is receiving more and more attention in the general population [15] and in KTRs as well [16,17]. Patients with ESRD often suffer from protein energy wasting, malnutrition, and sarcopenia [18], and shortly after KTX, they face sudden weight gain and multiple metabolic changes, which puts an additional burden on this vulnerable population [19,20].

Periodontitis is one of the most common chronic oral diseases in the adult population that affects supportive tooth tissues, causing their reduction and finally can lead to tooth loss [21]. Severe forms of periodontitis are associated with systemic chronic diseases such as CV, diabetes, and CKD responsible for a major number of deaths worldwide [22]. A significant body of evidence supports the association between severe forms of periodontitis and CKD, indicating that people with periodontitis have a higher prevalence of CKD [23]. Furthermore, in the systematic review of Dos Santos et al., it was indicated that periodontal status was associated with worsening graft function and systemic health among KTRs [24]. Considering the immunosuppressive therapy and comorbidities of this specific patient population, it is not clear whether periodontitis might present an additional risk to CKD in KTRs.

AGEs are stable compounds that accumulate on long-lived proteins. AGEs are formed by the reaction of proteins or lipids with aldose sugars and further molecular rearrangements. Age, the status of oxidative stress, inflammation, liver and kidney function, nutrition, and diet also influence AGE accumulation [25].

In CKD, accumulation of AGEs is attributed to impaired renal clearance, increased endogenous formation, and excess dietary intake [7,26]. Although plasmatic clearance of AGEs dramatically improves after KTX, their removal from slow-turnover tissues might be difficult to achieve and may contribute to this high CVD burden in this population of patients [27,28]. Some studies have linked tissue levels of AGEs with chronic allograft dysfunction and a poorer CVD outcome [29,30]. The magnitude of AGE contribution to the important cardiovascular risk in KTR is not clear.

The aim of the study was to evaluate carotid intima-media thickness (IMT) as a surrogate marker of atherosclerosis and also to analyze possible risk factors for carotid IMT in Dalmatian KTRs.

## 2. Materials and Methods

### 2.1. Study Design and Population

Kidney transplant recipients (KTRs, *n* = 93) older than 18 years of age, with functioning kidneys and no mobility difficulties, were included in this cross-sectional study conducted at the outpatient clinic of the Department of Nephrology and Dialysis, University Hospital of Split, Croatia, between July 2019 and October 2019. The study protocol was approved by the Ethics Committee of the University Hospital of Split, Croatia.

We excluded patients who met one of the following exclusion criteria: had an implanted pacemaker or cardioverter defibrillator, stents, or limb amputation; refused to participate in the study; did not undergo ultrasound or periodontal examination; had an active infection; or had active malignant disease.

### 2.2. Medical History, Clinical and Laboratory Parameters

By thorough examination of patients’ medical records, data on the existence and duration of primary chronic kidney disease, arterial hypertension, diabetes mellitus, and time of kidney transplantation (KTX), type, and duration of dialysis treatment before KTX were obtained.

Regarding laboratory parameters, all study participants underwent usual peripheral blood sampling, and they were asked to obtain a 24 h urine sample on the same day as the body composition and blood pressure measurement. We collected data on levels of urea (mmol/L), creatinine (mmol/L), uric acid (mmol/L), serum albumin (g/L), phosphates (mmol/L), C-reactive protein (CRP; mg/L), calcium (mmol/L), glucose (mmol/L), triglycerides (mmol/L), total cholesterol (mmol/L), low-density lipoprotein cholesterol (LDL) (mmol/L), hemoglobin (g/L), mean cellular volume (MCV), sodium (mmol/L), potassium (mmol/L), and eGFR using CKD-EPI (mL/min/1.73 m^2^). A complete blood count was obtained using a hematology analyzer (Advia 120, Siemens, Erlangen, Germany).

#### 2.2.1. Ultrasound Examination

All measurements were performed by two experienced radiologists (M.D.N., S.L.K.) who were blinded to the clinical data and periodontal status of the patient. Ultrasound evaluation of the carotid artery was performed in the B-mode technique using a high-resolution ultrasound scanner (LOGIQ S8, GE, Healthcare, Medical Systems, Waukesha, WI, USA) equipped with a 7–10 MHz linear transducer. The patient was examined in the supine position with the neck semiextended. After optimization of image quality, the scanning was started from the proximal part of the common carotid artery toward the bifurcation, followed by scanning the internal carotid artery. Dynamic images were stored for the evaluation of intima-media thickness (IMT), which was defined as the distance between the leading edges of the lumen interface and the media–adventitia interface at the far wall.

The points of measurement were 0.5 cm, 1 cm, and 2 cm distances from the bifurcation in the plaque-free area. The final carotid IMT was calculated as the mean of these six measurements (three on both sides). The increased value was defined as IMT ≥ 0.9 mm. The atheromatous plaque was considered as a focal wall thickening of at least 50% greater than that of the surrounding vessel wall.

The evaluation of quantitative ultrasound Doppler parameters included peak systolic velocity (PSV), end-diastolic velocity (EDV), and resistive index (RI) of both internal carotid arteries.

#### 2.2.2. Body Composition and Anthropometry Measurements

Body composition was assessed by bioelectrical impedance analysis (BIA) using an MC-780 Multi Frequency Segmental Body Analyzer (Tanita, Tokyo, Japan) for each participant. Data on muscle mass (kg), skeletal muscle mass (kg), skeletal muscle mass percentage (%), body mass (kg), fat mass (kg), fat mass percentage (%), fat-free mass (kg), and visceral fat were obtained. All participants were asked not to eat or drink excessively, exercise, or consume alcohol at least one day before the measurement. They also did not take any food or liquid for a minimum period of three hours prior to the measurement.

When it comes to anthropometric parameters, data on height, weight, body mass index (BMI), waist circumference (WC), mid-upper arm circumference (MUAC), and waist-to-height ratio (WHtR) were obtained for each study subject.

#### 2.2.3. Advanced Glycation End-Product (AGE) Measurement

AGEs were measured from the skin by skin autofluorescence (SAF) using a noninvasive desktop device (AGE Reader mu, Diagnostic’s Technologies BV, Groningen, The Netherlands). SAF is expressed in arbitrary units (AU) [31]. The device uses a UV-A light-emitting lamp and a built-in spectrometer to calculate SAF by dividing the excitation light by the emitted light. Before the measurement, the skin of each participant’s dominant forearm was cleaned with alcohol and placed on top of the device. All measurements were performed at a site of the skin with no visible abnormalities. Three consecutive measurements were performed for each study participant, and the mean value of SAF was calculated.

#### 2.2.4. Central Blood Pressure and Arterial Stiffness Measurement

The Agedio B 900 (IEM, Stolberg, Germany) device, which uses oscillometry technology, was used to assess central and peripheral blood pressure and arterial stiffness. Data on pulse wave velocity (PWV) (m/s), augmentation index (AiX) (%), peripheral systolic blood pressure (pSBP), peripheral diastolic blood pressure (pDBP), peripheral mean arterial pressure (pMAP), peripheral pulse pressure (pPP), central systolic blood pressure (cSBP), central diastolic blood pressure (cDBP), central mean arterial pressure (cMAP), and central pulse pressure (cPP) were obtained. After measuring the upper arm circumference, the right-sized cuff was selected and positioned according to the user manual. All measurements were performed with every participant sitting comfortably with their back and arm supported, feet flat on the ground, and legs not crossed. Participants were advised not to speak or move during the measurement.

#### 2.2.5. Periodontal Status Examination

The structured periodontal anamnesis based on the questionnaire was taken from all participants. The frequency of dental check-ups, personal and family history of periodontitis, smoking and oral hygiene habits, and clinical symptoms of periodontitis were recorded. A comprehensive periodontal examination was performed by an experienced periodontist (MR) using the UNC 15 mm periodontal probe (Aesculap, Tuttlingen, Germany). All clinical parameters measured on six sites were recorded: the full-mouth plaque score (FMPS), bleeding on probing (BOP), probing pocket depth (PPD), gingival recession (GR), and clinical attachment level (CAL). FMPS and BOP were expressed in percentages, whereas PPD, GR, and CAL were expressed in millimeters. The number of teeth, mean PD, mean CAL, BOP, FMPS, number of teeth with PD of 4, 5, and ≥6 mm, percent of sites with PD of 4, 5, and ≥6 mm, and number of teeth with interdental CAL of 1–2, 3–4, and ≥5 mm were periodontal variables assessed for each participant and included in the analysis.

Periodontal variables were considered in order to determine periodontal stages according to the new classification scheme, as proposed by Tonetti et al. [32]. Mild to moderate periodontitis included stages I and II, while severe periodontitis included stages III and IV.

### 2.3. Statistical Analyses

The normality of the data was assessed with the Shapiro–Wilk test. In cases of parametric distribution, numerical data were described with means and standard deviations (SDs), and with medians and interquartile ranges (IQRs) in cases of nonparametric distribution. Categorical data were described with numbers and percentages. To assess if there were any statistical differences between the groups of KTRs with increased IMT (atherosclerosis, IMT ≥ 0.9 mm) and those without (no atherosclerosis, IMT, 0.9 mm), the chi-square test was used for categorical data, T-test for parametric numerical data, and the Mann–Whitney U test for nonparametric numerical data. Spearman’s rank correlation was used to assess if there were any statistically significant correlation between IMT and measured numerical parameters. Finally, multivariate logistic regression analysis was performed to find predictors for atherosclerosis in KTRs, where all statistically significant variables identified from descriptive statistics and correlation analysis (with added gender information) were used as independent variables (named Full model). All these variables were then used as input for the Boruta algorithm [33] as a feature selection algorithm, which iteratively compares the importance of variables to keep. The selected variables were then checked for collinearity, and if any had a variance inflation factor (VIF) higher than 4, it was removed. These newly selected variables were used as independent variables in the second regression model (named Reduced model), and its output was used in the stepwise regression model with both forward and backward selection to identify the most important predictors for atherosclerosis in KTRs (named Final model). All regression models were mutually compared using the Akaike information criterion (AIC), while the quality of the models was assessed with the Hosmer and Lemeshow goodness-of-fit test. Results of logistic regressions were presented with odds ratios (ORs) and 95% confidence intervals (Cis). Statistically significant results were those with a value of *p* < 0.05. The entire statistical analysis was performed using the free software environment for statistical computing, R version 4.0.0 [34].

## 3. Results

**Figure 1:**
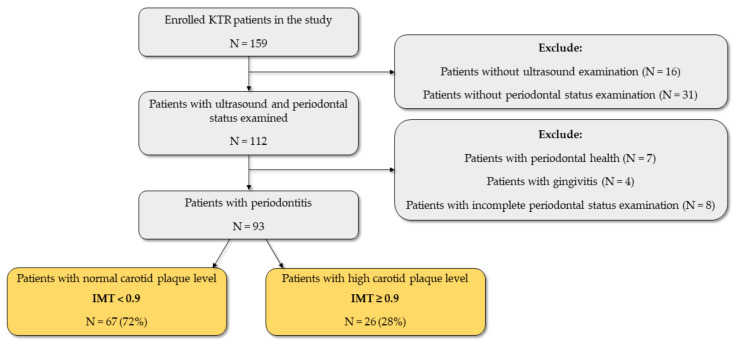
Flow diagram of the study. Abbreviations: KTR, kidney transplant recipient; IMT, intima-media thickness.

This study included 93 KTR patients. The exclusion criteria and number of excluded patients are presented in Figure 1. Data on basic characteristics, comorbidities, laboratory parameters, anthropometric, body composition, blood pressure, and ultrasound parameters, as well as periodontal status parameters, are shown in Table 1. To determine the significant level of IMT, we used an IMT cut-off of 0.9 mm. Sixty-seven (72%) KTRs had IMT < 0.9 mm, and 26 (28%) of them had IMT ≥ 0.9 mm. KTRs with IMT ≥ 0.9 were significantly older, had a lower level of total cholesterol, fat mass (kg), and end-diastolic velocity (EDV), and had fewer teeth. In addition, KTRs with IMT ≥ 0.9 mm had significantly higher values of pulse wave velocity (PWV) and resistive index (RI).

**Figure 2:**
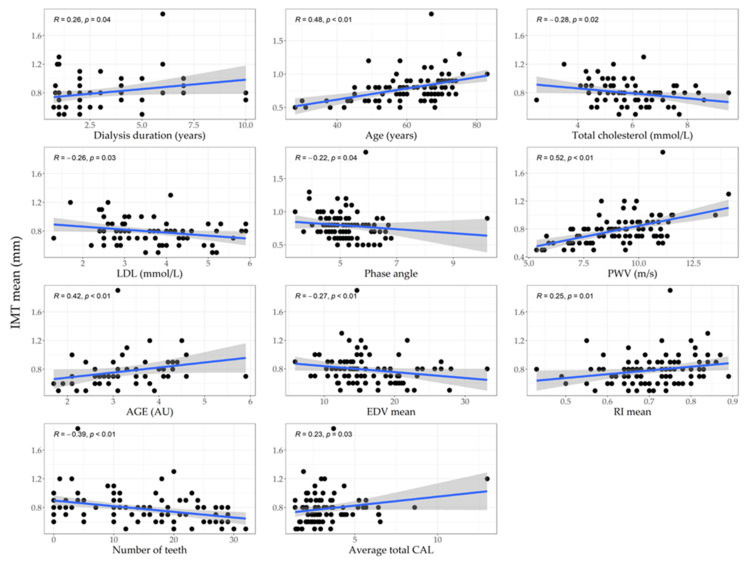
Correlations between IMT and measured parameters. Abbreviations: IMT, intima-media thickness; LDL, low-density lipoprotein cholesterol (mmol/L); PWV, pulse wave velocity (m/s); AGE, advanced glycation end-products (AU); EDV, end-diastolic velocity; RI, resistive index; CAL, clinical attachment loss.

Correlations between IMT and measured parameters are shown in Figure 2 (only statistically significant parameters are shown). Positive correlations between IMT and duration of dialysis, age, PWV, AGE, RI, and average total CAL were found. On the other hand, negative correlations between IMT and total cholesterol level, LDL, phase angle, EDV, and number of teeth were found.

Results of multivariate logistic regression models aiming to find predictors for atherosclerosis in KTRs are shown in Table 2. After selecting statistically significant variables from descriptive statistics and correlation analysis, 12 variables were used as independent predictors for atherosclerosis in KTRs. Sex was additionally added as an independent variable, as it could be a potential confounder. Only LDL and total cholesterol showed significant collinearity (VIF > 4, data not shown) in the Full model, while after feature selection with the Boruta algorithm, there were no issues with collinearity (Reduced model). The Boruta algorithm selected nine variables as important (dialysis duration, LDL, and phase angle were discarded). After applying a stepwise selection of both backward and forward selection, five variables were retained in the Final model. All three regression models showed good quality (Hosmer and Lemeshow goodness-of-fit test *p*-value > 0.05).

**Figure 3:**
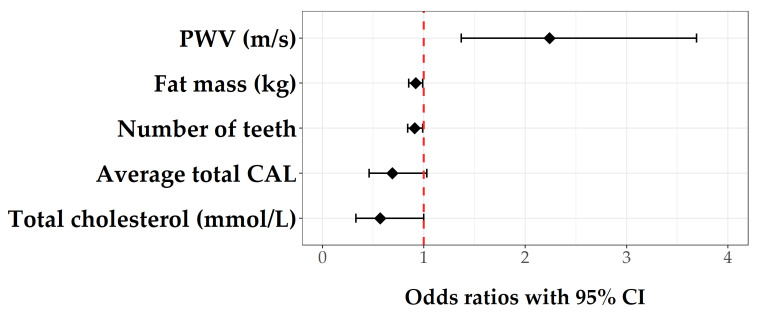
Most important predictors of atherosclerosis in KTRs (final model). Abbreviations: PWV, pulse wave velocity (m/s); CAL, clinical attachment loss; CI, confidence interval.

The final regression model showed that the greater odds of developing atherosclerosis in KTRs are associated with higher levels of PWV, lower fat mass, and fewer teeth. Average total CAL and total cholesterol levels were also selected to remain in the final model. However, these variables were not statistically significant (Figure 3). The total explained variance of the final model was 48.7%, with PWV alone contributing to 25.5%.

## 4. Discussion

To our knowledge, this is the first study that evaluated the associations of carotid IMT with AGEs, body mass composition, periodontal, and blood pressure parameters in KTRs.

The results of our study state that 27% of KTRs have IMT ≥ 0.9 mm and that those KTRs with IMT ≥ 0.9 mm were statistically older. It is well known that age is a major risk factor for atherosclerotic cardiovascular disease [35]. Additionally, in previous studies that evaluated IMT in KTRs, older age was associated with an increase in IMT [36,37]. In line with that finding is the positive correlation between IMT and age among all study subjects. Our results showed that those KTRs with IMT ≥ 0.9 mm had significantly lower fat mass (in kg) and lower cholesterol levels. Lower fat mass and a lower cholesterol level might be a reflection of protein energy wasting in KTRs. In line with our result, Mineoka et al. demonstrated a relationship between malnutrition and subclinical atherosclerosis in patients with type 2 diabetes [38]. Additionally, those KTRs with IMT ≥ 0.9 mm had significantly fewer teeth. Results from a previous study in the elderly population showed that the number of teeth is related to atherosclerotic plaque in the carotid arteries [39].

Therefore, we found a significant positive correlation between dialysis duration before KTR and IMT among all study subjects. In contrast to our study, no relationship between IMT and duration of dialysis treatment was found in a previous study in hemodialysis patients [40]. The possible explanation for this difference could be the larger number of participants in our study, and our participants were not treated just with hemodialysis before KTR.

Among the 112 KTRs with ultrasound and periodontal status examined in our study, 93 (83.08%) of them had periodontists. These results suggest a high prevalence of periodontitis in this population of patients, even higher than in patients with stage 5 CKD [41]. Moreover, the results of our study showed poor plaque control in both groups of KTRs. Considering that plaque is considered the most important etiologic risk factor for periodontitis and thus a source of microorganisms that can enter the bloodstream from periodontal tissues with inflammatory changes, it would be important to improve the oral hygiene habits of KTRs. Recently, it has been suggested that the use of toothpastes containing probiotics or paraprobiotics can significantly reduce periodontal inflammation [42]. Mouthwashes based on probiotics could also be considered in place of the commonly prescribed chlorhexidine antiseptics to avoid the known side effects of long-term use of chlorhexidine, such as tooth discoloration [43]. The use of mouthwashes is particularly important for plaque control in the elderly with limited manual dexterity [44]. However, paraprobiotics should be considered more for use under KTRs because they are inactive microorganisms that pose less risk of use in this compromised patient population. Further studies should be conducted on this topic. A positive correlation between CAL and IMT was found, although there was no difference in CAL among the two groups according to IMT categories, possibly due to the small number of participants. However, KTRs with IMT ≥ 0.9 mm were significantly older and had fewer teeth, which has an impact on mastication and consequently on fat mass. The possible reasons for these findings were out of the scope of this study and warrant further exploration. Since our study showed the big prevalence of periodontitis among KTRs and the association with carotid IMT, periodontal care would be recommended as a part of whole medical management for this vulnerable patient population. Those KTRs with a higher carotid IMT value had significantly fewer teeth and a significantly higher value of CAL. Additionally, it is important to note that our results showed that those periodontal parameters are one of the most important predictors of carotid IMT in this population of KTRs [9].

Additionally, KTRs with IMT ≥ 0.9 mm had statistically higher levels of oscillometric determined PWV as an indirect parameter of arterial stiffness [45]. In addition to that, higher levels of EDV and RI were noticed in KTRs with IMT ≥ 0.9 mm. All these findings could be explained by Bernoulli’s principle of blood flow, which defines the higher blood velocity in a blood vessel with a lower diameter and lower elasticity [46].

Interestingly, we found no statistically significant differences between KTRs regarding carotid IMT thickness in parameters of central or peripheral blood pressure. Some studies suggest higher levels of blood pressure in people with higher levels of arterial stiffness in KTRs [9,47], and although we have observed this trend, it was not statistically significant in our sample size. It is important to highlight that KTR patients are under constant nephrologist supervision and great care is taken for their blood pressure regulation, which is one of the possible explanations for results in this population of patients.

It is well known that carotid IMT is a surrogate marker for the presence and progression of atherosclerosis [48]. Our results showed a significant positive correlation between PWV and IMT in this population of patients. Therefore, our final regression model showed that one of the greater odds for the carotid IMT in this population of KTRs is PWV. Both carotid IMT and PWV were thought to result from accumulative exposure to CV factors over the life course, explaining their association with CV morbidity and mortality in the general population [49]. Furthermore, data suggest that PWV predicts mortality in KTRs [50]. Data also suggest that there is no significant change in aortic PWV in the first year post-transplantation, in contrast to the majority of studies demonstrating the progression of arterial stiffness in patients with CKD over the same period [51]. Results from a recent pilot study that investigated changes in vascular abnormalities over time in stable KTRs showed the worsening of vascular structure and function where PWV and IMT increased during 6 months of follow up [51].

Additionally, our results showed a significant positive correlation between AGEs and carotid IMT among all study subjects. AGEs are involved in the progression of atherosclerosis and some chronic diseases, such as chronic renal failure, Alzheimer’s disease, and diabetes mellitus [52,53,54,55]. Additionally, AGEs have also been associated with endothelial dysfunction and early vascular aging [40]. Patients with CKD are affected by an increased level of chronic inflammation and oxidative stress, and these conditions may contribute to the increased production of AGEs [56,57]. The result of a study with KTRs showed a lower mean AGE value than in our study population (2.8 vs. 3.26). A possible explanation for these results could be that our population of KTRs is older (62 years vs. 52 years) [58].

The results showed a significant negative correlation between phase angle and carotid IMT. Phase angle is a bioimpedance analysis parameter that indirectly shows body cell mass and is associated with muscle mass, strength, physical performance, quality-of-life scale, and hospitalization-free survival in maintenance HD patients [59]. Additionally, phase angle was significantly associated with mortality during the 8-year follow-up period of KTRs [60]. These results suggest the possible correlation between nutritional status and carotid IMT in KTRs.

On the other hand, our final regression model showed that one of the greater odds for the higher value of carotid IMT in this population of KTRs is lower fat mass. This finding is in contrast with a previous study conducted by Mohsen et al., who evaluated atherosclerotic changes in the carotid artery following KTR and found that IMT measures significantly correlated with age and BMI [12]. A possible explanation for our findings could be the influence of malnutrition inflammation atherosclerosis syndrome and protein energy wasting on IMT and atherosclerosis during dialysis treatment in our population of patients. Additionally, hemodialysis patients have indicated reverse associations of obesity with all-cause and CV mortality in many survival studies [61]. Therefore, low values for BMI are associated with increased mortality, whereas higher values for BMI were found to be protective and associated with improved survival in dialysis patients. This paradox has been referred to as “reverse epidemiology” [62]. In contrast, a study on hemodialysis patients has shown that atherosclerosis was correlated with a high body fat percentage for female hemodialysis patients, but not for males [63]. It is important to note that recent data suggest that underweight and severe obesity at transplantation are associated with a significantly increased risk of graft loss and patient death. A target BMI at KTR is 22–27 kg/m^2^ [64].Our population of KTRs had a BMI of 26.87 kg/m^2^. To clarify this association between fat mass and atherosclerosis risk in KTRs, future studies with a larger number of KTRs are needed in the prospective study design.

Another important factor is vitamin D, a relevant element for CV risk and nutritional status in KTRs [65], which we, unfortunately, did not take into consideration when conducting our study.

Our study has some limitations, which primarily come from the cross-sectional design, which, as such, prevents us from any causal conclusions. Possible limitations to the measurement of AGEs in our study may include endogenous factors present in the skin that absorb emission light, such as melanin in dark-skinned subjects, acute illness, and strenuous exercise associated with glycoxidative stress, exogenous factors such as diet, and, for example, the use of skincare products. Considering the limitations, measurement with the AGE reader is a validated method with 5% to 6% intraobserver variation in repeated autofluorescence measurements within a day [66]. Prospective studies and randomized controlled trials (RCTs) with larger samples and in a multicenter setting are needed to investigate the causal relationship between periodontal disease and IMT in this patient population.

## 5. Conclusions

The results from this study suggest possible associations between AGEs and nutritional and periodontal status with carotid IMT as a surrogate marker of atherosclerosis in KTRs. Nutritional intervention for those patients who have lost teeth is a great challenge. It should include individualized dental and nutritional counseling. Therefore, consumption of soft foods should be advised, and if that nutritional intervention does not meet the needs, oral nutritional supplementation should be implemented. All in all, more attention should be paid to the nutritional and dental management of those KTRs with tooth loss.

## Figures and Tables

**Table 1:** Basic characteristics and differences according to intima-media thickness (IMT) in KTRs.

	All (N = 93)	IMT < 0.9 mm (N = 67)	IMT ≥ 0.9 mm (N = 26)	*p*
Time since transplantation (years), median (IQR)	4.5 (6.62)	4 (6.25)	6 (6.5)	0.206
Dialysis type, N (%)				
PD	35 (38.04)	26 (39.39)	9 (34.62)	0.686
HD	51 (55.43)	35 (53.03)	16 (61.54)	
PD + HD	6 (6.52)	5 (7.58)	1 (3.85)	
Dialysis duration (years), median (IQR)	2 (3)	2 (2)	3.5 (2.25)	0.130
Age (years), median (IQR)	62 (14)	59 (15)	69.5 (7.75)	**<0.001**
Sex, N (%)				
Women	43 (46.24)	34 (50.75)	9 (34.62)	0.243
Men	50 (53.76)	33 (49.25)	17 (65.38)	
Presence of arterial hypertension, N (%)				
No	12 (12.9)	10 (14.93)	2 (7.69)	0.556
Yes	81 (87.1)	57 (85.07)	24 (92.31)	
Presence of diabetes mellitus, N (%)				
No	73 (78.49)	54 (80.6)	19 (73.08)	0.609
Yes	20 (21.51)	13 (19.4)	7 (26.92)	
Smoking status, N (%)				
Nonsmoker	41 (48.81)	29 (47.54)	12 (52.17)	0.690
Former smoker	27 (32.14)	19 (31.15)	8 (34.78)	
Smoker	16 (19.05)	13 (21.31)	3 (13.04)	
Presence of chronic kidney disease, N (%)				
eGFR > 60 mL/min/1.73 m^2^	27 (30.34)	18 (28.12)	9 (36)	0.638
eGFR < 60 mL/min/1.73 m^2^	62 (69.66)	46 (71.88)	16 (64)	
**Laboratory parameters**				
Alb (g/L), median (IQR)	42 (4.75)	42 (5)	42 (4.25)	0.878
Ca (mmol/L), median (IQR)	2.43 (0.17)	2.44 (0.18)	2.42 (0.12)	0.711
CRP (mg/L), median (IQR)	2.5 (3.78)	2.6 (3.75)	1.8 (2.7)	0.406
E, median (IQR)	4.71 (0.65)	4.69 (0.65)	4.78 (0.66)	0.546
GUP (mmol/L), median (IQR)	5.3 (0.88)	5.34 (0.82)	5.19 (1.04)	0.483
Hb (g/L), median (IQR)	135.24 (15.97)	133.67 (16.57)	139.24 (13.82)	0.140
K (mmol/L), mean (SD)	4.11 (0.49)	4.13 (0.5)	4.03 (0.44)	0.375
Total cholesterol (mmol/L), mean (SD)	5.96 (1.27)	6.2 (1.23)	5.42 (1.2)	**0.016**
Creatinine (mmol/L), median (IQR)	126 (59)	127.5 (54.5)	117 (50)	0.246
LDL (mmol/L), median (IQR)	3.6 (1.03)	3.75 (1.01)	3.25 (1.04)	0.056
MCV (fL), mean (SD)	87.89 (5.6)	87.67 (5.88)	88.44 (4.93)	0.566
Na (mmol/L), median (IQR)	141 (3)	141 (3)	141 (3.25)	0.984
P (mmol/L), median (IQR)	1 (0.22)	1.01 (0.23)	0.99 (0.15)	0.263
Tgl (mmol/L), median (IQR)	1.8 (1.25)	1.9 (1.1)	1.65 (0.79)	0.152
Uric acid (mmol/L), median (IQR)	394 (76.25)	400 (81)	387 (60)	0.485
Urea (mmol/L), median (IQR)	9 (6)	9.15 (5.47)	8.9 (4.9)	0.338
eGFR (mL/min/1.73 m^2^), median (IQR)	47.1 (27.4)	46.35 (30.17)	50.9 (26.9)	0.235
**Anthropometric parameters**				
BMI (kg/m^2^), mean (SD)	26.84 (4.02)	27.07 (4.36)	26.29 (3.07)	0.412
Middle upper arm circumference (cm), median (IQR)	30 (7)	30 (5)	27 (8.5)	0.568
Waist circumference (cm), mean (SD)	101 (12.24)	100.95 (12.89)	101.17 (10.22)	0.948
WHtR, mean (SD)	0.58 (0.07)	0.58 (0.07)	0.59 (0.06)	0.927
**Body composition parameters**				
Fat mass (kg), median (IQR)	20.24 (8.49)	21.43 (8.97)	17.43 (6.6)	**0.044**
Fat mass (%), mean (SD)	24.57 (8.58)	25.7 (8.88)	21.94 (7.31)	0.061
Fat-free mass (kg), median (IQR)	60.2 (18.25)	57.2 (16.5)	64.6 (17.93)	0.547
Visceral fat, mean (SD)	9.72 (3.82)	9.4 (4.08)	10.46 (3.09)	0.239
Muscle mass (kg), median (IQR)	57.2 (17.4)	54.3 (15.7)	61.35 (17.05)	0.547
Skeletal muscle mass (kg), median (IQR)	32 (11.4)	31.1 (11.9)	35.1 (11.12)	0.650
Skeletal muscle mass (%), median (IQR)	40.78 (6.3)	40.27 (6.67)	41.98 (5.25)	0.246
Phase angle, median (IQR)	5.1 (0.9)	5.1 (0.8)	4.95 (0.85)	0.134
Bone mass (kg), mean (SD)	3.03 (0.54)	3.01 (0.53)	3.08 (0.55)	0.572
Trunk visceral fat (kg), mean (SD)	10.63 (5.01)	11.25 (5.28)	9.2 (4.04)	0.081
**Blood pressure parameters**				
pSBP (mHg), mean (SD)	134.14 (19.52)	132.25 (18.87)	139.17 (20.71)	0.140
pDBP (mHg), mean (SD)	86.31 (12.85)	85.8 (12.06)	87.67 (14.97)	0.546
pMAP (mHg), mean (SD)	109.27 (14.23)	108.16 (13.67)	112.5 (15.62)	0.231
pPP (mHg), mean (SD)	50.88 (14)	49.2 (12.27)	55.79 (17.57)	0.063
cSBP (mHg), mean (SD)	127.95 (17.6)	126.07 (16.45)	133.4 (20.03)	0.100
cDBP (mHg), mean (SD)	86.98 (12.58)	86.66 (12)	87.93 (14.38)	0.692
cMAP (mHg), mean (SD)	100.64 (13.15)	99.8 (12.57)	103.08 (14.76)	0.327
cPP (mHg), mean (SD)	37.51 (11.73)	36.21 (10.53)	41.26 (14.29)	0.089
HR, mean (SD)	71.47 (11.19)	71.48 (11.11)	71.44 (11.65)	0.988
PR, median (IQR)	1.83 (0.35)	1.83 (0.33)	1.83 (0.46)	0.852
AIx, median (IQR)	19.75 (19)	19.5 (19)	23 (17)	0.865
PWV (m/s), mean (SD)	9.05 (1.75)	8.55 (1.6)	10.38 (1.43)	**<0.001**
AGE (AU), mean (SD)	3.26 (0.88)	3.16 (0.89)	3.58 (0.81)	0.130
**Ultrasound parameters**				
IMT mean, mean (SD)	0.8 (0.2)	0.7 (0.2)	1 (0.18)	**<0.001**
PSV mean, mean (SD)	57.11 (13.33)	56.88 (12.27)	57.72 (15.99)	0.786
EDV mean, median (IQR)	15 (6.55)	15.85 (6.25)	13.68 (2.9)	**0.007**
RI mean, mean (SD)	0.71 (0.09)	0.69 (0.09)	0.75 (0.08)	**0.008**
**Periodontal status parameters**				
Number of teeth, median (IQR)	14 (16)	16 (13.5)	10 (13.75)	**0.024**
Dental plaque (%), median (IQR)	87.5 (40)	87 (40)	90 (40)	0.611
Bleeding (%), median (IQR)	10 (26.75)	13 (24)	5 (34)	0.457
Average pocket depth, median (IQR)	1.98 (0.84)	2.08 (0.86)	1.91 (0.79)	0.340
Average total CAL, median (IQR)	2.86 (1.34)	2.75 (1.41)	2.97 (1.21)	0.602
Reason for tooth loss, N (%)				
Periodontitis	39 (41.94)	24 (35.82)	15 (57.69)	0.092
Other	54 (58.06)	43 (64.18)	11 (42.31)	
Periodontitis stage, N (%)				
I + II (mild)	48 (51.61)	39 (58.21)	9 (34.62)	0.070
III + IV (severe)	45 (48.39)	28 (41.79)	17 (65.38)	

Abbreviations: IMT, intima-media thickness; PD, peritoneal dialysis; HD, hemodialysis; eGFR, estimated glomerular filtration rate using CKD-EPI (mL/min/1.73 m^2^); BMI, body mass index (kg/m^2^); WHtR, waist-to-height ratio; Alb, serum albumin (g/L); Ca, calcium (mmol/L); CRP, C-reactive protein (mg/L); E, erythrocyte count; GUP, fasting glucose (mmol/L); Hb, hemoglobin (g/L); K, potassium (mmol/L); LDL, low-density lipoprotein cholesterol (mmol/L); MCV, mean cellular volume (fL); Na, sodium (mmol/L); P, phosphates (mmol/L); Tgl, triglycerides (mmol/L); pSBP, peripheral systolic blood pressure (mHg); pDBP, peripheral diastolic blood pressure (mHg); pMAP, peripheral mean arterial pressure (mHg); pPP, peripheral pulse pressure (mHg); cSBP, central systolic blood pressure (mHg); cDBP, central diastolic blood pressure (mHg); cMAP, central mean arterial pressure (mHg); cPP, central pulse pressure (mHg), HR, heart rate (beat/minute); PR, peripheral resistance; Aix, augmentation index; PWV, pulse wave velocity (m/s); AGE, advanced glycation endproducts (AU); IMT, intima-media thickness; PSV, peak systolic velocity; EDV, end-diastolic velocity; RI, resistive index; CAL, clinical attachment loss.

**Table 2:** Multivariate logistic regression for identification of predictors for atherosclerosis in KTRs.

Predictor	OR	95% CI	*p*
**Full model (Nagelkerke’s *R*^2^:** **0.566, AIC: 91.83)**			
Dialysis duration (years)	1.29	0.9–1.85	0.172
Age (years)	1.1	0.93–1.3	0.278
Sex (men)	3.6	0.62–20.87	0.153
Total cholesterol (mmol/L)	0.57	0.07–4.43	0.591
LDL (mmol/L)	1.33	0.12–14.85	0.815
Fat mass (kg)	0.93	0.84–1.02	0.142
Phase angle	2.9	0.75–11.2	0.123
PWV (m/s)	2.49	1.05–5.92	**0.038**
AGE (AU)	0.51	0.18–1.48	0.218
EDV mean	0.87	0.68–1.11	0.266
RI mean	0	0–33.84	0.139
Number of teeth	0.87	0.77–0.99	**0.035**
Average total CAL	0.54	0.3–0.97	**0.038**
**Reduced model (after feature selection with Boruta; Nagelkerke’s *R*^2^: 0.513, AIC: 89.26)**			
Sex (men)	3.45	0.8–14.91	0.098
Age (years)	1.02	0.89–1.18	0.765
Total cholesterol (mmol/L)	0.67	0.38–1.2	0.183
Fat mass (kg)	0.95	0.88–1.03	0.194
PWV (m/s)	2.4	1.02–5.64	**0.045**
EDV mean	0.89	0.72–1.11	0.313
RI mean	0	0–204.34	0.257
N teeth	0.88	0.79–0.98	**0.024**
Average total CAL	0.7	0.45–1.06	0.095
**Final model (after stepwise selection; Nagelkerke’s *R*^2^: 0.487, AIC: 83.88)**			
Total cholesterol (mmol/L)	0.57	0.33–1	0.051
Fat mass (kg)	0.92	0.85–0.99	**0.033**
PWV (m/s) *	2.24	1.37–3.69	**0.001**
Number of teeth	0.91	0.84–0.99	**0.032**
Average total CAL	0.69	0.46–1.03	0.072

* PWV contributes to 25.5% of total explained variance. Abbreviations: LDL, low-density lipoprotein cholesterol (mmol/L); PWV, pulse wave velocity (m/s); AGE, advanced glycation end-products (AU); EDV, end-diastolic velocity; RI, resistive index; CAL, clinical attachment loss.

## Data Availability

Raw data are available at the corresponding author mail: josiparadic1973@gmail.com.
